# One-Step Fabrication of UiO-66/PVDF/PGE and MOF-199/PVDF/PGE Electrode for High-Performance Supercapacitors

**DOI:** 10.1021/acsomega.4c11211

**Published:** 2025-03-28

**Authors:** Ozay Eroglu, H. Sevval Dere, Afike Ayca Ozen, Sema Aslan, Siti Nadiah Abdul Halim, Ugur Erkarslan, Hulya Kara Subasat

**Affiliations:** † Department of Energy, Molecular Nano-Materials Laboratory, Mugla Sıtkı Kocman University, Mugla 48000, Turkey; ‡ Department of Chemistry, Mugla Sıtkı Kocman University, Muğla 48000, Turkey; § Department of Chemistry, Faculty of Science, Universiti Malaya, Kuala Lumpur 50603, Malaysia; ∥ Department of Physics, Molecular Nano-Materials Laboratory, Mugla Sıtkı Kocman University, Mugla 48000, Turkey

## Abstract

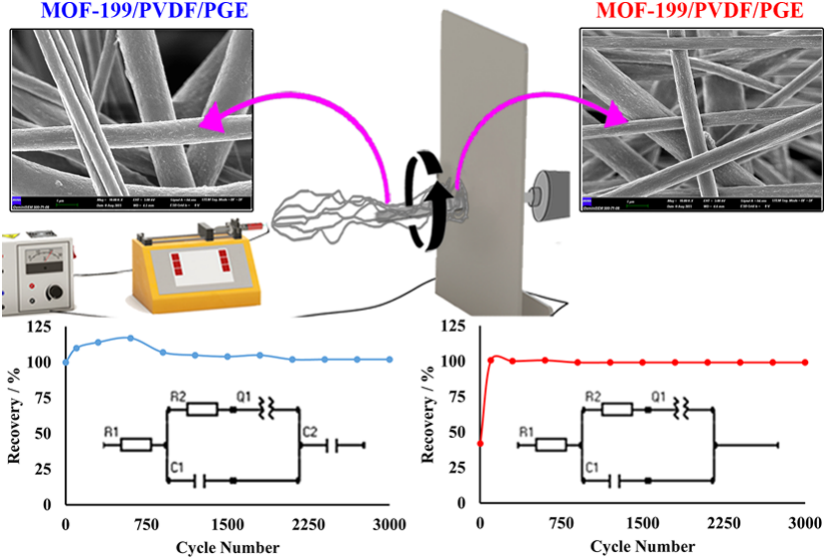

With the increasing importance of energy storage technologies, the demand for supercapacitors combining high energy density with fast reversible rechargeability is increasing. However, conventional multistep synthesis methods increase the production costs and limit the practical application of these technologies. To solve this problem, we developed two innovative electrodes, UiO-66/PVDF/PGE and metal–organic framework (MOF)-199/PVDF/PGE. These electrodes produced with a one-step electrospinning technique provide a cost-effective solution by simplifying the fabrication process and reducing costs. The successful incorporation of MOFs into the polymer matrix was confirmed by Fourier transform infrared (FTIR) and X-ray diffraction (XRD) analyses, and a homogeneous nanofiber morphology was observed by scanning electron microscope (SEM) imaging. Thermogravimetric analysis (TGA) and dynamic mechanical analysis (DMA) analyses showed significant improvements in the thermal and structural stability of the composites. The electrochemical properties were analyzed in detail by cyclic voltammetry (CV) and electrochemical impedance spectroscopy (EIS) methods. The electrospun UiO-66/PVDF/PGE electrode demonstrated a high specific capacitance of 1619.26 F/g with exceptional cycling performance at 1 A/g current density, while the MOF-199/PVDF/PGE electrode achieved a value of 933.19 F/g. Both electrodes maintained 99.16 and 102.04% of their initial capacitance after 3000 cycles, respectively, exhibiting outstanding stability for long-term energy storage applications. These results demonstrate that UiO-66/PVDF/PGE and MOF-199/PVDF/PGE are promising as scalable, high-performance, and cost-effective electrode materials for supercapacitor technologies.

## 1 Introduction

The development of energy storage devices is a significant challenge in the 21st century to meet the needs of modern society.^1^ Supercapacitors (SCs) have garnered immense attention due to their high-power density, fast charge/discharge speed, and excellent cycle stability.^2^ The performance of these devices is largely determined by the design of their electrode materials, which are critical components in energy storage systems. Despite advancements, achieving the simultaneous optimization of high energy density, long cycle life, and fast charge/discharge capability remains a challenge.^3,4^ Researchers are focusing on innovative electrode material structures to address this issue and improve the performance of supercapacitors, which stand out as a promising energy storage technology.

Among the various candidate materials, metal–organic frameworks (MOFs), with their high surface areas, tunable pore structures, and crystalline architectures, are promising materials for energy storage applications.^5^ Notable examples, such as MOF-199 (Cu-BTC) and UiO-66 (Zr-MOF), exhibit redox-active metal centers and high porosity, making them particularly suitable for supercapacitor electrodes.^6,7^ Since the charge transfer capabilities are enhanced proportionally with the electrochemical current harvest by using MOFs therefore the electrical energy storage is facilitated.^8^ Furthermore, solutions like MOF-based porous carbons via pyrolysis and MOF-polymer composites have been developed to enhance conductivity and ion transport.^6,9^ MOF-199, with a surface area exceeding 2000 m^2^/g and redox-active Cu^2+^ sites, offers high specific capacitance and thermal stability. Carbonized derivatives and polymer composites, such as those with polyaniline (PANI), further improve its conductivity and capacitance, achieving up to 766 C/g.^10^

Similarly, UiO-66-based composites have shown remarkable performance in energy storage applications. For instance, UiO-66/ZrO_2_ composites achieved specific capacitance values up to 913.8 F/g at a current density of 1 A/g, with a retention of 86% stability after 3000 cycles.^11^ Comparatively, hybrids such as polyaniline (PANI)/UiO-66 composites demonstrated a capacitance of 647 F/g and 91% stability after 5000 cycles.^9^ These findings underscore the competitive performance of UiO-66-based materials in terms of specific capacitance and long-term cycling stability, reinforcing their potential for advanced energy storage solutions. Additional enhancements, such as coatings with polydopamine (PDA) and polypyrrole (PPy), further optimize ion transport and conductivity, showcasing the potential of MOF-polymer hybrids in advanced energy storage solutions.^7,12^

On the other hand, the electrospinning process is considered to be one of the most recent simple and inexpensive methods for the production of nanofibers (NFs) accompanied by a high specific surface area from different morphological nanostructured materials.^13−16^ Electrospun one-dimensional (1D) nanofibers stand out as excellent platforms for electrode materials due to their adjustable nanoscale dimensions, distinctive porous structures, affordability, and straightforward fabrication process.^17^ Based on these versatile properties of nanofibers, our previous studies have leveraged the unique properties of one-step nanofiber-based materials to develop advanced high-performance supercapacitor electrodes. Innovative modifications of nanofiber/carbon-based materials in these studies revealed significant advancements in performance. TiO_2_ nanoparticles combined with polyacrylonitrile (PAN) nanofibers produced a synergistic effect, achieving a specific capacitance of 156.00 F/g.^18^ Similarly, a nanofiber network incorporating liquid crystals and Coumarin 500 demonstrated enhanced electrochemical properties, attaining a specific capacitance of 410.60 F/g and exhibiting remarkable stability over 2500 cycles.^19^ These findings highlight the potential of tailored nanofiber networks in achieving high-performance energy storage solutions.

Here, we developed two innovative electrodes, UiO-66/PVDF/PGE and MOF-199/PVDF/PGE, for supercapacitor applications. While previous studies have investigated the applications of UiO-66 and MOF-199 individually or in combination with other materials,^9,20^ our work presents two new composite structures combining these MOFs with poly(vinylidene difluoride) (PVDF). This approach harnesses the strengths of both MOFs and the polymer matrix, creating a synergy that enhances the electrochemical performance, stability, and flexibility of the electrode materials. Another innovation of our work lies in the one-step electrospinning method, which simplifies the fabrication while offering improved performance compared to previous studies that required more complex fabrication processes.^21^ By combining MOF-199 and UiO-66 with PVDF, a flexible polymer matrix known for its ionic conductivity properties, the aim is to develop high-performance and durable electrode materials that can be applied to various energy storage devices by utilizing the synergistic effects of MOFs and PVDF. This approach not only exploits the energy storage potential of MOFs, but also overcomes their inherent drawbacks, providing a green future path for supercapacitor technologies.

## 2 Experimental Section

### 2.1 Materials and Measurements

All chemicals and solvents were of analytical grade, commercially obtained and used as received without any additional purification. Poly(vinylidene fluoride) (PVDF (C_2_H_2_F_2_)_n_), *M*_w_: 534.000, Copper(II) nitrate trihydrate (Cu(NO_3_)_2_.3H_2_O), Hydrochloric acid (HCl), Dimethylformamide (DMF), Ethanol, Acetone, Potassium chloride (KCl), Potassium hydroxide (KOH), Potassium hexacyanoferrate(II).trihydrate (K_4_[Fe(CN)_6_].3H_2_O), reference electrode (Ag/AgCl filled with 0.1 M KCl standard solution supplied by Ionode firm), Pt foil (Ionode) (counter electrode) and the Sodium Phosphate Dibasic Heptahydrate (Na_2_HPO_4_·7H_2_O) and Sodium Phosphate Monobasic Monohydrate (NaH_2_PO_4_·H_2_O) salts used for the Phosphate-buffered saline (PBS) were purchased from Sigma-Aldrich. Zirconium(IV) chloride (ZrCl_4_), 1,3,5-benzenetricarboxylic acid (H_3_BTC) and 1,4-benzodicarboxylic acid (H_2_BDC) were purchased from Acros Organics. Pencil graphite electrode (PGE, Micro, 0.9 mm) was purchased from a local market.

Infrared spectra of the samples were collected using a PerkinElmer Spectrum 400 Fourier transform infrared (FTIR)/FT-FIR spectrometer (USA). The crystallinity of the MOF materials was analyzed with a PANalytical X’Pert HighScore diffractometer equipped (Rigaku Smartlab) with primary monochromatic high-intensity Cu–Kα radiation (λ = 1.54184 nm), scanning over a range of 5–40°. The morphology of the nanofibers was examined using a field emission scanning electron microscope (FESEM, ZEISS GEMINI 500). Nanofiber diameters were measured from SEM images using ImageJ software (NIH, Bethesda, MD). Thermogravimetric analysis (TGA) of the nanofiber samples was performed with a PerkinElmer Thermal Analyzer under a nitrogen atmosphere (2.5 bar, 10 mL/min flow rate), with a heating rate of 10 °C/min over a temperature range of 30–700 °C. Dynamic mechanical analysis (DMA) was conducted using the TA Instruments Q800 DMA device on samples measuring 20 mm in length and 3 mm in width. A crosshead speed of 5 mm/min was applied, with a force increment of 0.1 N/min. Electrochemical experiments were performed using a triple-electrode system connected to a Gamry 1010E Potentiostat-Galvanostat. The setup included a platinum foil as the counter electrode, an Ag/AgCl electrode as the reference electrode, and the nanofiber-coated PGE as the working electrode.

### 2.2 Synthesis

#### 2.2.1 Synthesis of UiO-66

The synthesis of UiO-66 (Zr_6_O_4_(OH)_4_(BDC)_6_), [BDC = 1,4-benzenedicarboxylic acid (C_8_H_6_O_4_)] was performed following a previously reported method with some modifications.^22^ Initially, H_2_BDC (0.116 g, 0.7 mmol) was dissolved in 10 mL of DMF at room temperature under constant stirring until a clear solution was obtained. Simultaneously, ZrCl_4_ (0.117 g, 0.5 mmol) was dissolved in a mixture of DMF and HCL at a 5:1 ratio. The solution was stirred until fully dissolved. The H_2_BDC solution was then added dropwise to the ZrCl_4_ solution, and the resulting mixture was transferred to a round-bottom flask. The mixture was subjected to reflux at 85 °C with stirring for 23 h. Upon completion of the reaction, a white precipitate formed, which was collected via centrifugation. The solid was washed thoroughly with DMF (3 times) to remove unreacted residues, followed by drying in a hot air oven at 70 °C for 24 h. The process yielded 0.198 g of a white powder with a yield of 93%. Anal. Calc. for UiO-66: (Zr_6_O_4_(OH)_4_(C_8_H_6_O_4_)_6_): Calc.: C: 32.43; H: 1.36%. Found: C: 32.38; H: 1.37%.

#### 2.2.2 Synthesis of MOF-199

The synthesis of MOF-199 (Cu_3_(BTC)_2_) [BTC = 1,3,5-benzenetricarboxylic acid (C_9_H_3_O_6_)] was carried out following a previously reported method with some modifications.^22^ H_3_BTC (0.3045 g, 3.0 mmol) was dissolved in a minimal amount of ethanol at room temperature, while Cu(NO_3_)_2_·3H_2_O (0.114 g, 1.1 mmol) was dissolved in deionized water under the same conditions. Both solutions were stirred separately until completely clear. The two solutions were then combined in a single beaker and stirred at temperatures ranging from 70 to 100 °C for 15–30 min. During this process, a light blue precipitate gradually formed, indicating the successful coordination of copper ions with H_3_BTC. The reaction mixture was allowed to cool to room temperature, and the precipitate was left to settle naturally. The resulting light blue solid was collected by filtration, thoroughly washed with ethanol and deionized water (3 times each) to remove unreacted residues, and then dried in an oven at 70 °C overnight. The final product was a light blue powder weighing 0.252 g, corresponding to an 85.8% yield. Anal. Calc. for MOF-199: (Cu_3_(C_9_H_3_O_6_)_2_): Calc.: C: 42.10; H: 1.18%. Found: C: 42.12; H: 1.17%.

#### 2.2.3 Preparation of MOFs/PVDF/PGE Nanofiber Electrodes

PVDF/MOF-based solutions were prepared by first dissolving PVDF in a solvent mixture of DMF and acetone (7:3 v/v) at 50 °C under continuous stirring for 24 h to obtain a 16 wt % PVDF solution. Subsequently, 0.12 g of ground MOFs (UiO-66 or MOF-199) was added to 5 mL of the prepared PVDF solution, yielding a 15 wt % MOF concentration. The resulting mixtures were homogenized using a magnetic stirrer, followed by treatment in an ultrasonic bath to ensure uniform dispersion.

**Figure 1:**
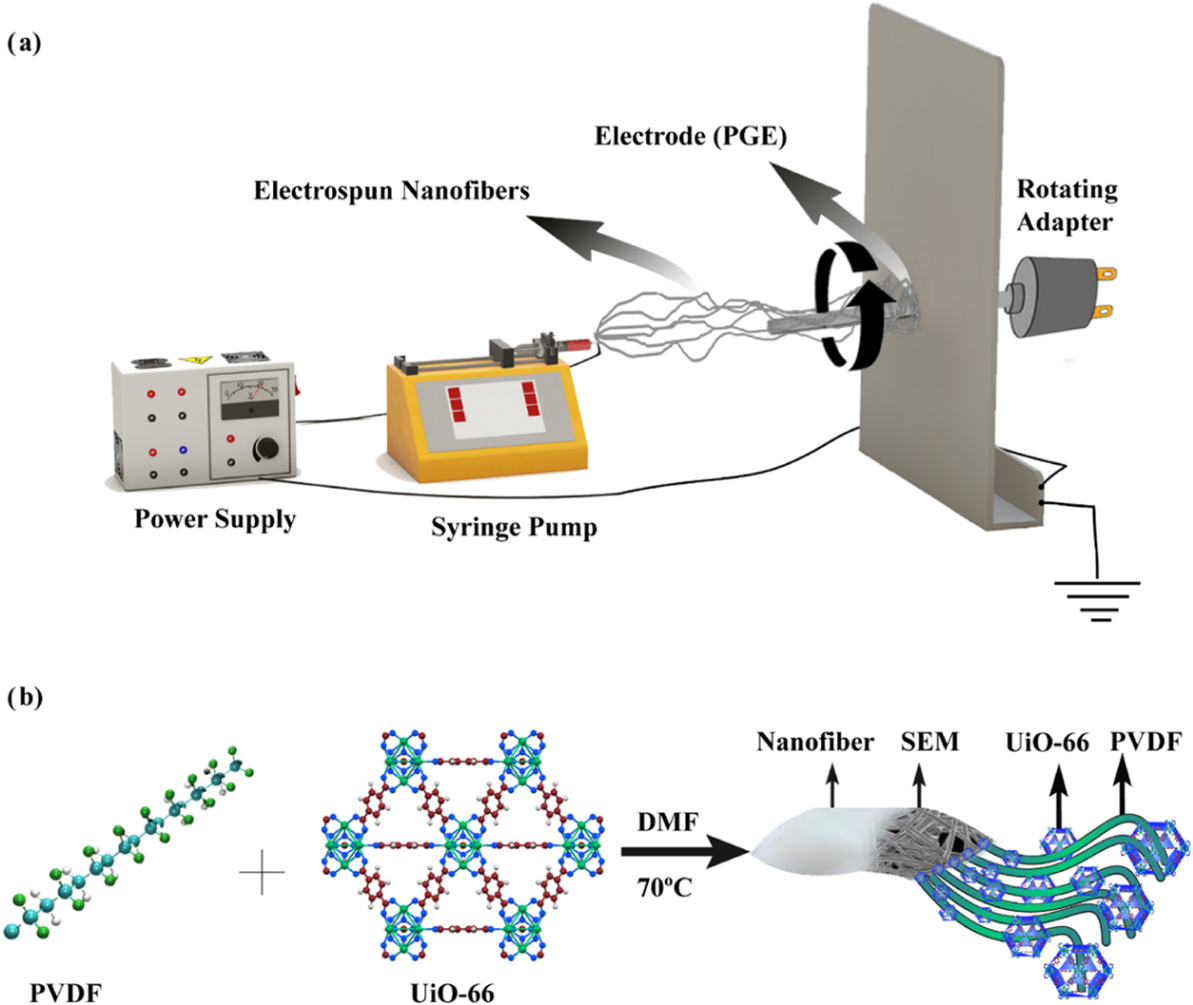
Schematic representation of (a) the electrospinning process (b) the nanofiber structure of UiO-66/PVDF. The photos were created by one of the authors.

MOF/PVDF/PGE electrodes were fabricated using a single-step electrospinning method previously developed by our group,^19^ employing the Spingenix SG100 electrospinning device. During fabrication, the electrodes were deposited on an aluminum foil-coated collector plate, positioned perpendicular to the injector via a rod-shaped adapter (Figure 1a). This approach integrates the synthesis and coating of composite materials into a single step, eliminating conventional multistep fabrication processes. Consequently, it simplifies production, reduces costs, and enhances scalability. The electrospinning parameters were optimized to ensure a homogeneous distribution of MOFs (UiO-66 and MOF-199) within the PVDF matrix and effective coating on the PGE surface. This ensured the consistent fabrication of MOF/PVDF nanofiber electrodes with uniform fiber distribution and optimal surface morphology, essential for improved electrochemical performance.

For the electrospinning process, the above-prepared PVDF/MOF-based solutions were loaded into 5 mL plastic syringes and extruded through an 18 G needle tip using a syringe pump operated at 20 kV. Nanofiber deposition was conducted on a rotating rod-shaped PGE at flow rates of 1.25 mL/min for UiO-66/PVDF and 1.40 mL/min for MOF-199/PVDF. The distance between the syringe tip and the collector was maintained at 20 cm throughout the process.

### 2.3 Electrochemical Measurements

Cyclic voltammetry (CV) and electrochemical impedance spectroscopy (EIS) were employed to conduct electrochemical measurements on PGE and nanofiber-coated composite electrodes. The experiments were conducted using 0.1 M KCl containing 0.05 M PBS solution (pH 7.4) as the supporting electrolyte, with the responses recorded in a 0.005 M K_4_[Fe(CN)_6_]·3H_2_O probe solution containing PBS. This probe system, comprising K_4_[Fe(CN)_6_]·3H_2_O, K_3_[Fe(CN)_6_]·3H_2_O, or their combination, is widely employed as a redox probe in electrochemical studies.^23^ The same electrolyte system was used for both CV and EIS measurements. The experimental setup included the electrode under investigation as the working electrode, with an Ag/AgCl electrode (filled with 0.1 M KCl, Ionode) serving as the reference and a Pt foil electrode (Ionode) as the counter electrode. CV measurements were performed within a potential range of −1.0 to +1.0 V, employing scan rates from 5 to 250 mV/s. For EIS measurements, a frequency range of 10^–1^–10^–4^ Hz was used, with 0.1 M KOH solution serving as the electrolyte medium.

The specific capacitance (*C*_s_) (F/g) of composite electrodes were determined using established equations.^19^ In these calculations, m represents the mass of the electroactive material on the electrode surface (mg) (0.00249 g for MOF-199 and 0.002535 g for UiO-66), Δ*V* is the applied charge–discharge potential (*V*), *S* denotes the scan rate (mV/s), and *I* indicate the current value (*A*) obtained from the electrode.
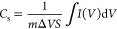
1

## 3 Results and Discussion

### 3.1 FTIR Studies

**Figure 2:**
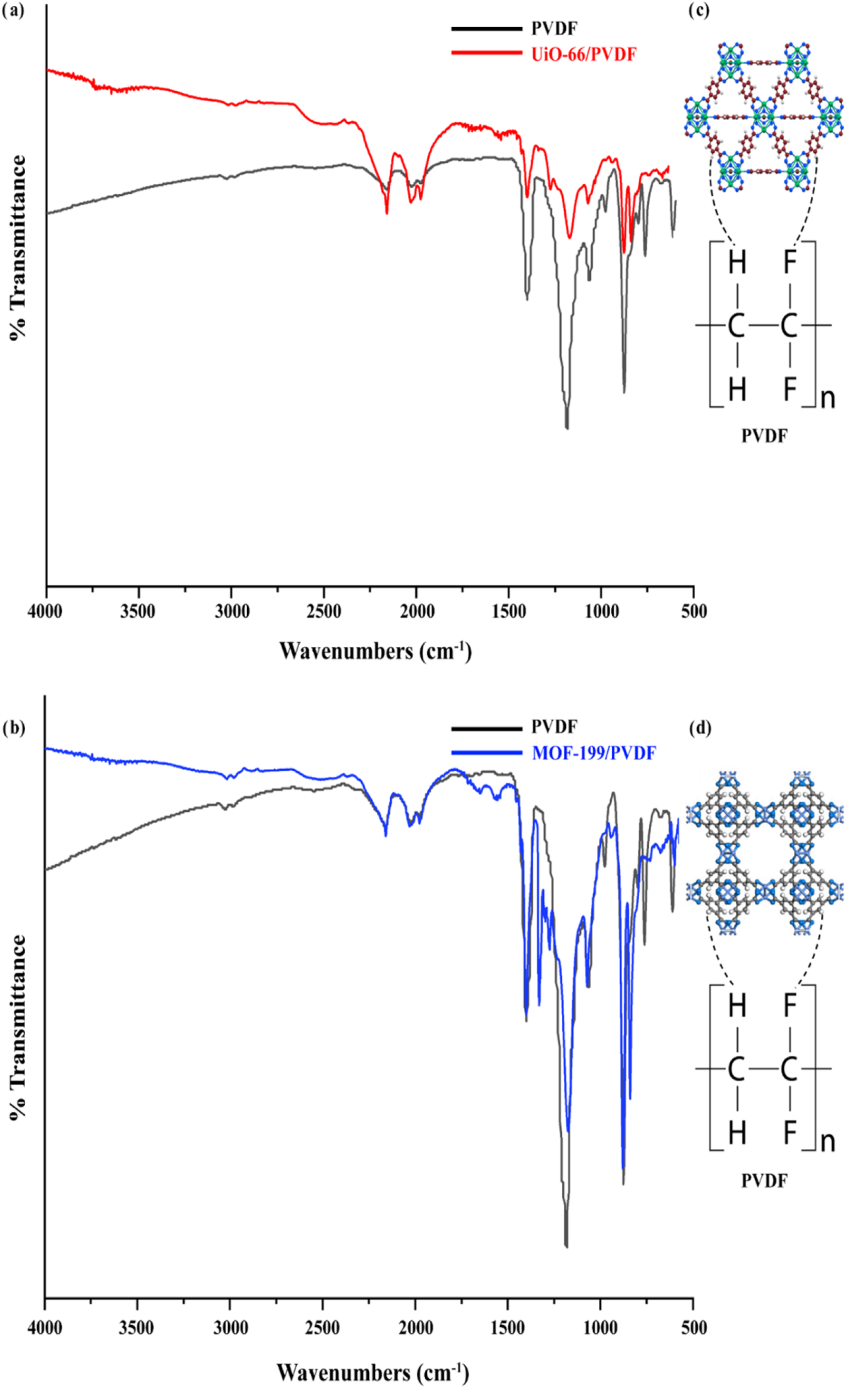
(a) FTIR spectra of PVDF and MOF-199/PVDF, (b) PVDF and UiO-66/PVDF. Intermolecular H bond interaction in (c) UiO-66/PVDF and (d) MOF-199/PVDF.

The FT-IR spectra of PVDF, MOF-199/PVDF, and UiO-66/PVDF composites are shown in Figure 2, with detailed wave numbers for PVDF, UiO-66, MOF-199, and their corresponding composites summarized in Table 1. In pure PVDF, characteristic peaks appear at 1180 cm^–1^, corresponding to −CF_2_ stretching vibrations, and at 843, 876, and 1060 cm^–1^, representing C–H vibrations associated with the α, β, and γ crystalline phases, respectively.^24−26^ For UiO-66, peaks observed at 1540 and 1400 cm^–1^ are attributed to C–C stretching in the aromatic compound and C–O stretching vibrations in the carboxylate group of BDC, respectively. Additionally, a peak at 748 cm^–1^ indicates Zr–O bonding. These findings align well with previously reported FTIR spectra of UiO-66.^27,28^ Similarly, in MOF-199, a notable peak at 1640 cm^–1^ is attributed to carboxylate group stretching vibrations coordinated to copper ions. The bands at 1342 and 1436 cm^–1^ reflect the vibrations of carboxylate groups in BTC, indicating nearly bidentate behavior of the COO moiety. Furthermore, a peak at 740 cm^–1^ corresponds to Cu–O bond vibrations. The results are in good agreement with previously reported FTIR spectra of MOF-199.^6,29^ In the UiO-66/PVDF and MOF-199/PVDF composites, the FTIR spectra exhibit overlapping absorption bands from both the respective MOFs and PVDF without the emergence of new peaks. This indicates that the interactions between the MOFs and PVDF are primarily physical rather than chemical, preserving the structural integrity of the MOFs within the composites. These findings are consistent with previous studies.^30^ No significant peak shifts were observed for UiO-66/PVDF and MOF-199/PVDF, although weak hydrogen bonding or other intermolecular forces may occur due to functional groups present in both components.

**Table 1:** IR Stretching Wavenumbers (cm^–1^) of PVDF, MOF-199, MOF-199/PVDF, UiO-66, UiO-66/PVDF

assignments	PVDF	UiO-66	UiO-66/PVDF	MOF-199	MOF-199/PVDF
*v*(C–C)		1540	1541		
*v*(C–O)		1400	1403		
*v*(C=O)				1640	1648
*v*_asym_(COO−)				1342	1330
*v*_sym_(COO−)				1436	1400
*v*(CF_2_)	1180		1174		1170
*v*(C–H) (α, β, γ)	843, 876, 1060		840, 878, 1073		837, 872, 1070
Zr–O		748	742		
Cu–O				740	746

The integration of MOF-199 and UiO-66 into a PVDF matrix results in a novel class of advanced materials that combines the superior properties of MOFs, such as high surface area, chemical stability, and hydrophilicity, with the flexibility and processability of PVDF. MOF-199 enhances energy storage capacity by providing abundant charge storage sites, while UiO-66 offers thermal stability, chemical robustness, and uniformly distributed micropores, facilitating ionic mobility and improving the operational durability of the composite. Additionally, intermolecular interactions contribute to the formation of continuous and stable MOF/PVDF structures. Specifically, the O–H groups in UiO-66 and C–F groups in PVDF are likely to facilitate weak hydrogen bonding, enhancing the composite’s stability (Figure 2c). Similarly, the carboxylate groups in MOF-199 and the C–F groups in PVDF contribute to improved compatibility and stability through weak hydrogen bonding (Figure 2d). This synergy results in composite materials with exceptional mechanical strength, enhanced hydrophilicity, and superior electrochemical performance, paving the way for significant advancements in high-performance supercapacitors.^31,32^

### 3.2 SEM Analysis

**Figure 3:**
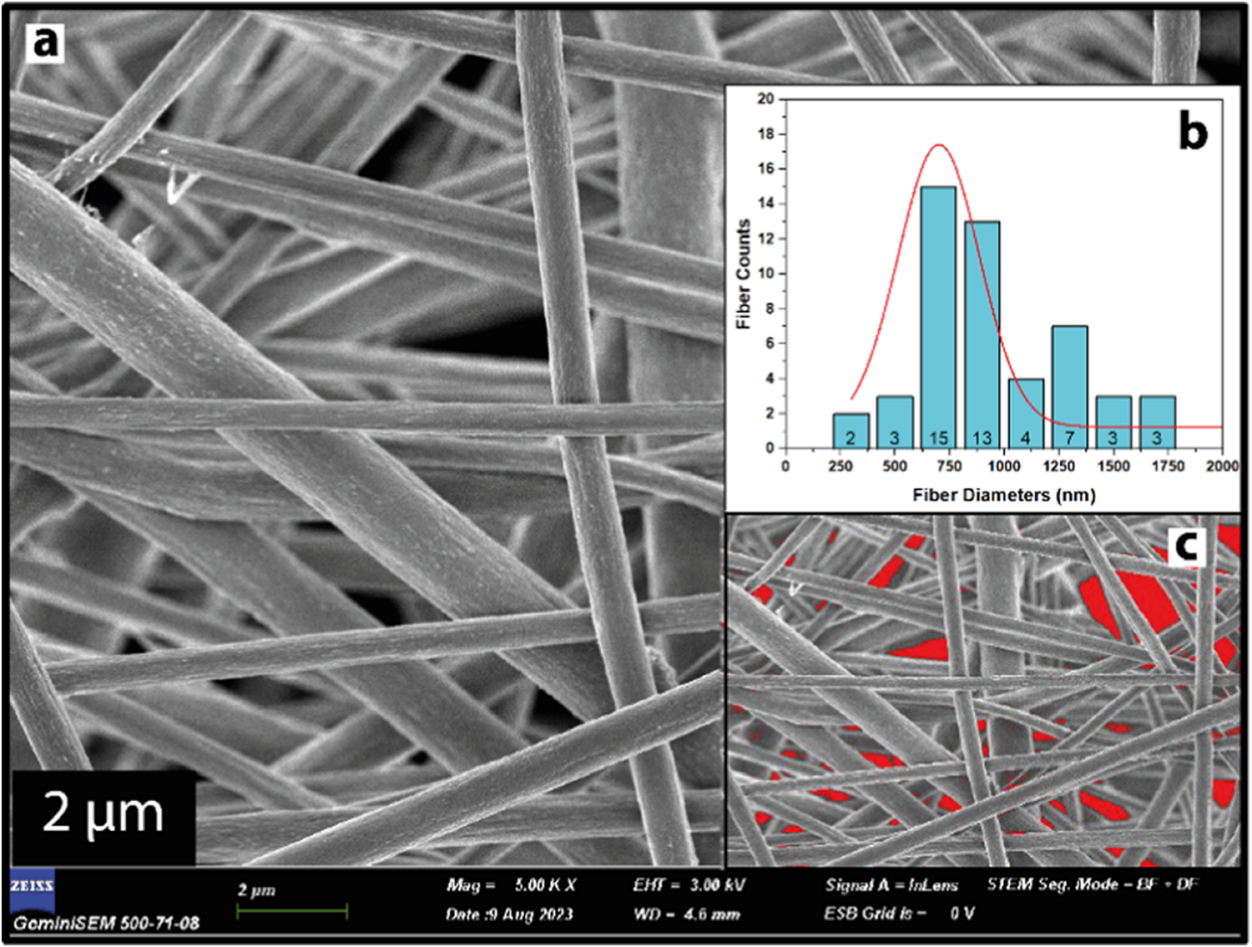
(a) SEM image of UiO-66/PVDF (scale bar, 2 μm) (b) fiber diameter distributions, (c) imageJ porosity distributions for UiO-66/PVDF nanofibers.

**Figure 4:**
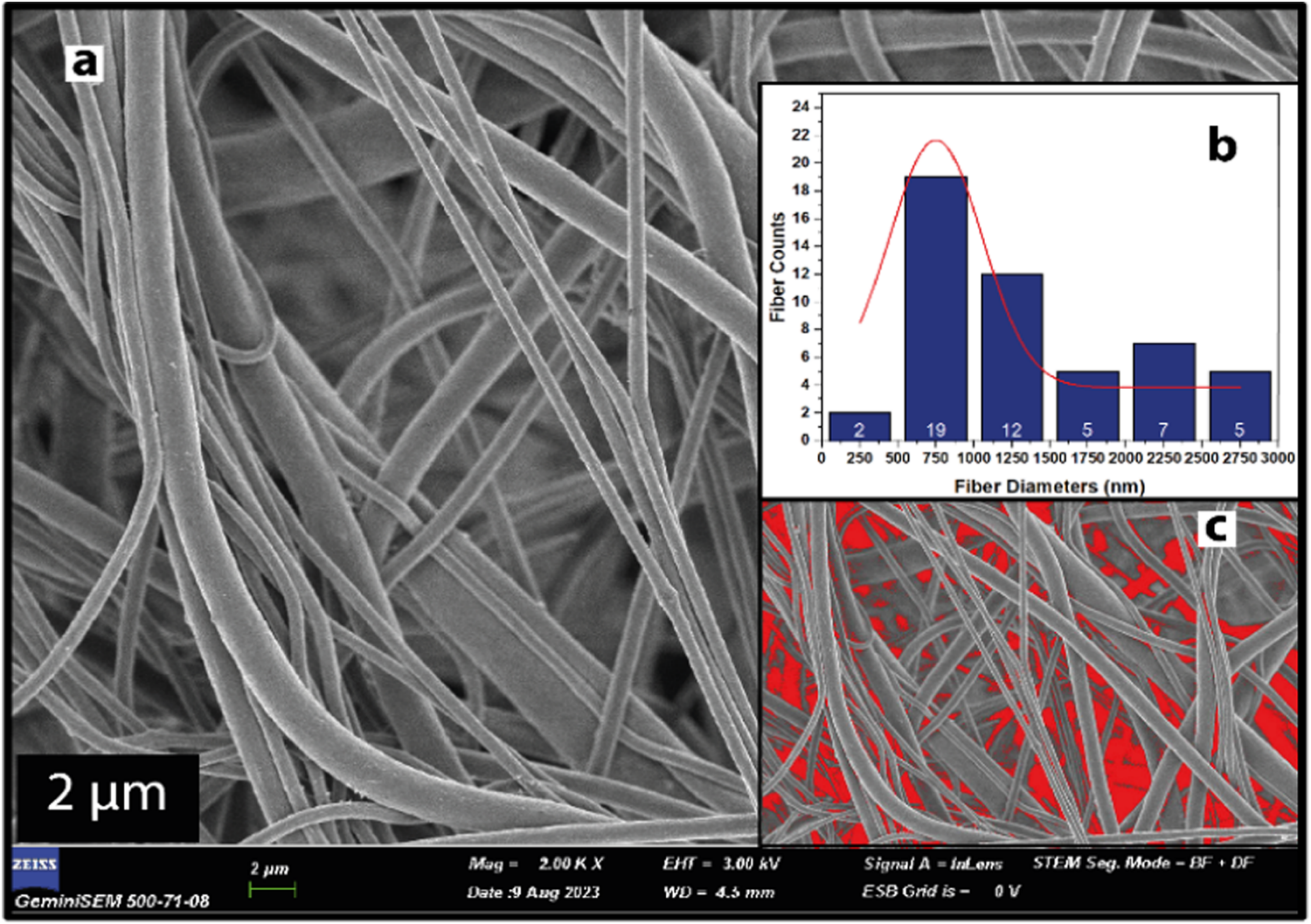
(a) SEM image of MOF-199/PVDF (scale bar, 2 μm) (b) fiber diameter distributions, (c) imageJ porosity distributions for MOF-199/PVDF nanofibers.

Figures 3a and 4a display FESEM images of UiO-66/PVDF and MOF-199/PVDF, respectively. The absence of beads in all fibers indicates uniformity throughout. Fiber thickness distributions were analyzed using ImageJ software on SEM images at 10 000× magnification. Each scatter plot in Figures 3b and 4b represents at least 50 different fibers, showing thickness distributions for UiO-66/PVDF and MOF-199/PVDF, respectively. SEM images were also used for porosity calculations. The average diameter for UiO-66/PVDF and MOF-199/PVDF was found to be 700 and 750 nm, respectively. Porosity measurements revealed void percentages of 66.62% for UiO-66/PVDF and 59.04% for MOF-199/PVDF, as illustrated in Figures 3c and 4c. UiO-66/PVDF exhibited higher porosity compared to MOF-199/PVDF. Bead formation usually occurs when electrospinning conditions, such as high surface tension or insufficient polymer solution viscosity, cause the jet to become unstable, resulting in droplets rather than continuous fibers. Since no bead formation was observed in our samples, it appears that the conditions used were suitable for maintaining fiber integrity, which is desirable for supercapacitor performance.^33^ Electrospinning parameters such as precursor viscosity, net charge density of the jet, feed rate, and precursor surface tension have been suggested to influence bead formation in electrospun fibers.^34−37^

### 3.3 Thermal Properties

**Figure 5:**
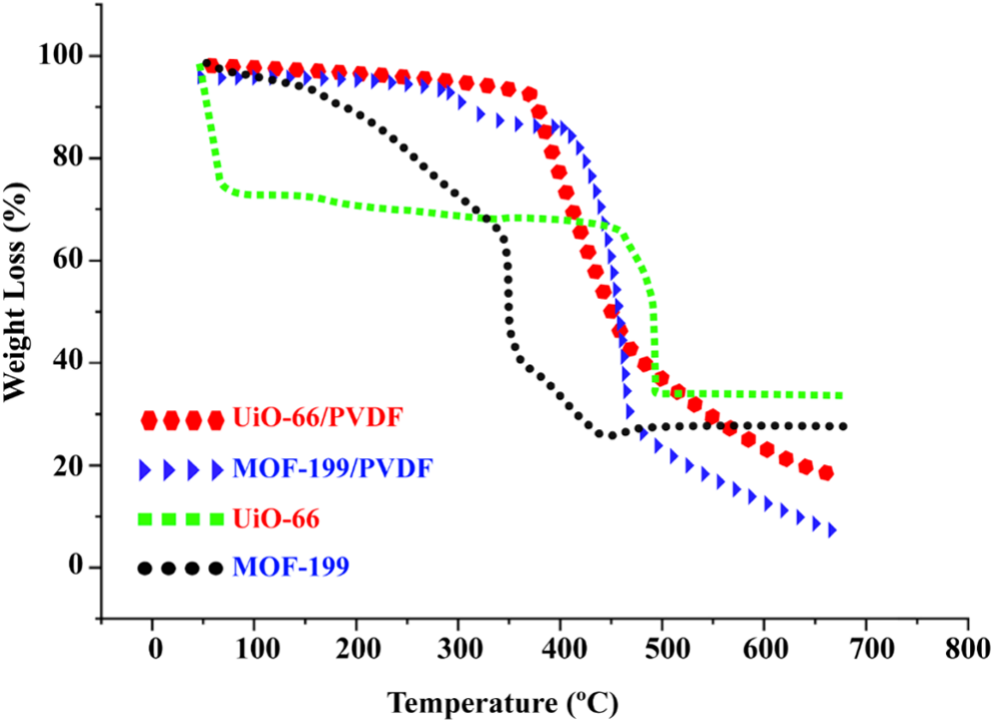
TGA measurements of UiO-66/PVDF, MOF-199/PVDF nanofibers, and UiO-66 and MOF-199.

TGA results provide a comparative evaluation of the thermal stability of pure metal–organic frameworks (MOFs) and their polymer matrix (PVDF) composites (Figure 5). The data indicate that pure UiO-66 and MOF-199 exhibit significant weight loss beginning around 300 °C, suggesting limited thermal stability under elevated temperature conditions. In contrast, the UiO-66/PVDF/PGE and MOF-199/PVDF/PGE composites show high improvement in thermal stability due to the incorporation of MOFs into the PVDF polymer matrix. The higher thermal stability of the composites is especially obvious for the MOF-199/PVDF/PGE sample, which still exhibits less weight loss at higher temperatures than its unmodified counterpart. This should definitely prove the role of the PVDF matrix in maintaining the structural integrity of the MOFs under thermal stress. On the other hand, the higher weight loss observed in UiO-66/PVDF and MOF-199/PVDF samples above 500 °C compared to pure UiO-66 and MOF-199 is thought to be most likely due to the thermal decomposition of the PVDF matrix. In the analyses performed in nitrogen atmosphere, PVDF decomposes in the range of 400–600 °C, causing an additional weight loss.^38^ In addition, physical or chemical interactions between MOFs and PVDF matrix may affect the thermal stability of MOFs and lead to a faster decomposition.^39^ Finally, the residual weight of pure MOFs may be higher due to the formation of stable metal oxides,^40^ but this effect becomes less pronounced in composites due to the decomposition products of PVDF. The incorporation of PVDF into MOFs appears to affect the thermal degradation of the composite.^38,41^ In summary, the results highlight that when metal–organic frameworks (MOFs) are incorporated into polymer matrices, not only their functional characteristics are retained but also their stability in terms of thermal degradation is significantly improved by going for such composites, which are potential strong contenders for applications at high temperatures^11,42,43^

### 3.4 Mechanical Analysis

**Figure 6:**
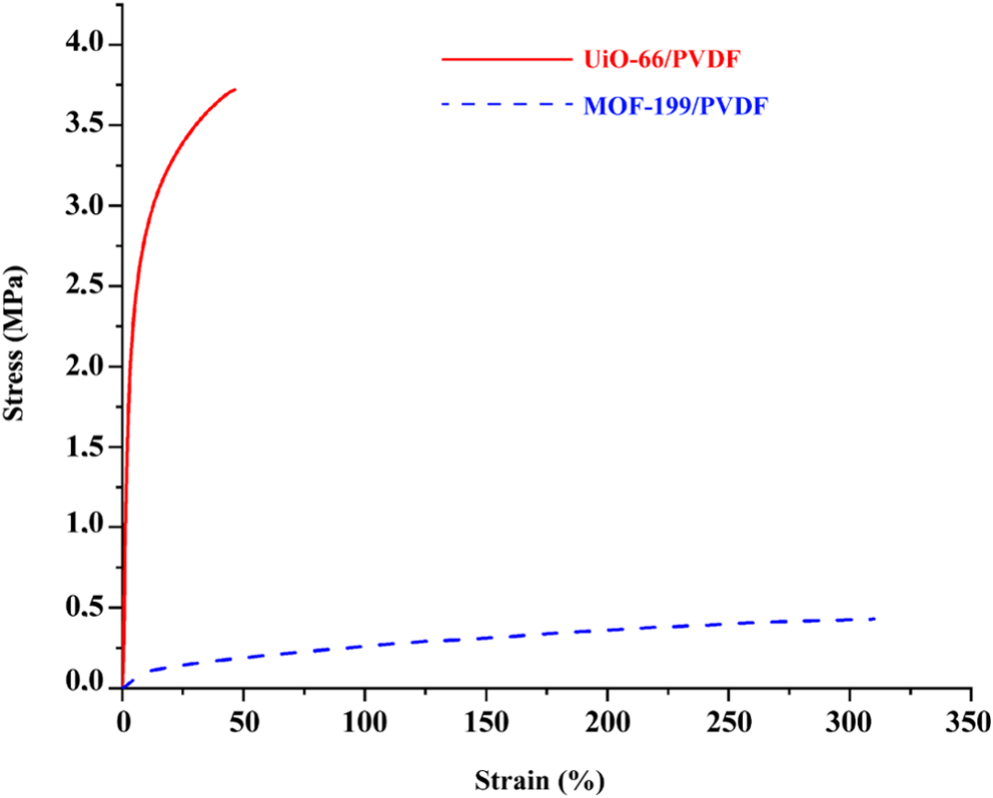
Stress–strain curves of UiO-66/PVDF and MOF-199/PVDF nanofibers.

The mechanical properties of MOFs/PVDF nanofiber composites were examined by measuring the stress–strain, which revealed the remarkably different mechanical performances between UiO-66/PVDF and MOF-199/PVDF nanofibers. As presented in Figure 6, UiO-66/PVDF nanofibers exhibited high mechanical strength with a maximum stress value of 3.7718 MPa, while their strain percent was relatively low at 46.34%. This observed behavior can be attributed to the rigid structure of UiO-66, which provides better structural stability but hinders the elongation due to its reduced flexibility.^22,44^ While the stress value of this MOF-199/PVDF nanofiber was only 0.4299 MPa, the strain percentage was 309.4%. This is a quite high value of strain considering the nature of MOF-199.^45,46^ Most likely, the flexibility and ductility of MOF-199, combined with the PVDF matrix, could have made this enhanced strain behavior possible. The addition of MOF-199 into the PVDF matrix seems to maintain its natural flexibility and at the same time allows for sufficient mechanical stability.^20,47^ Taken together, these results are in line with previous studies showing that the incorporation of MOFs into polymer matrices can effectively tune mechanical properties by changing the type of MOF and its interaction with the polymer.^48^

### 3.5 Electrochemical Performance

**Figure 7:**
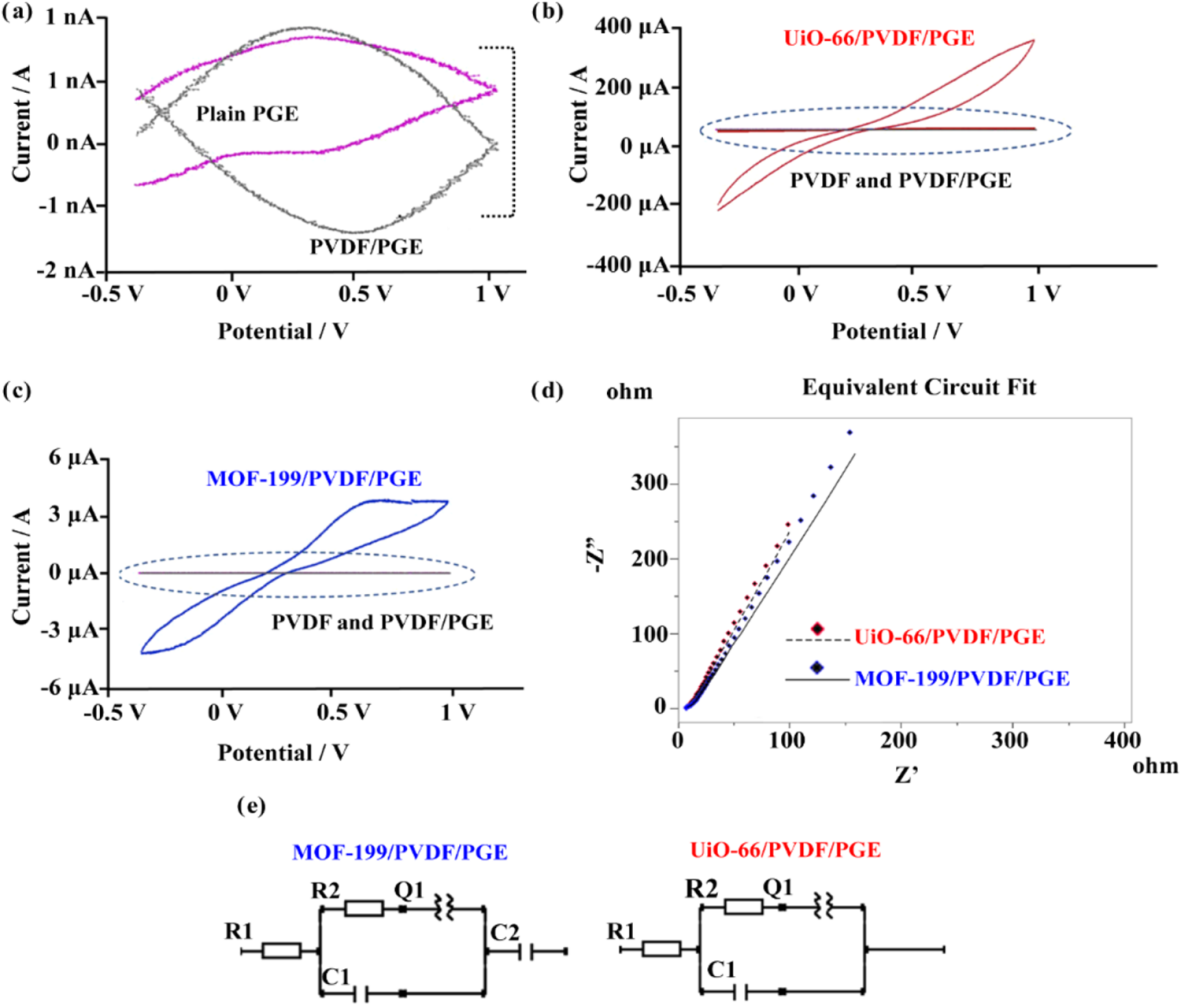
Electrochemical characterizations of developed electrodes. CV curves of the samples at 100 mV/s scan rate. (a) PGE and PVDF/PGE (b) UiO-66/PVDF–PGE (c) MOF-199/PVDF–PGE (d) Nyquist plot of the UiO-66/PVDF/PGE and MOF-199/PVDF–PGE (e) equivalent circuit of MOF-199/PVDF–PGE and UiO-66/PVDF/PGE.

To investigate the electrochemical properties of UiO-66 and MOF-199 on the PVDF-coated PGE electrode surfaces for supercapacitor applications, a range of electrochemical tests were conducted. The study focused on cyclic voltammetry (CV) and electrochemical impedance spectroscopy (EIS), analyzing the CV voltammograms and Nyquist plots of UiO-66/PVDF/PGE and MOF-199/PVDF/PGE electrodes (Figure 7). Comparative analysis of the CV voltammograms (Figure 7b,7c) highlights a substantial current increase upon incorporating UiO-66 and MOF-199 into the PVDF nanofiber structure. While the voltammograms of PGE and PVDF/PGE showed currents at the 2 μA level (Figure 7a), UiO-66/PVDF/PGE increased the current to 200 μA (Figure 7b). Similarly, doping with MOF-199 resulted in a current increase to 3 μA. These findings indicate that UiO-66 exhibits greater electroactivity than MOF-199. The observed enhancements in current between UiO-66/PVDF/PGE and MOF-199/PVDF/PGE electrodes, compared to the PVDF/PGE electrode, can be attributed to the host effect of the metallic centers and the rotational effect of the aromatic carbon–oxygen-aromatic cycles in the UiO-66 and MOF-199 molecules. These effects facilitate efficient electron transfer from the electrode surface.^49,50^ This electron transfer occurs within the hydrogen-bond-rich network of PVDF nanofibers, primarily between donor oxygen groups and central metallic groups.^51^ Such electron wiring and tunneling effects have been extensively employed in electrochemistry to enhance electron diffusion.^52−54^ Faradaic and nonfaradaic biosensor studies have leveraged these effects using conductive polymers and nanoparticle composites. Commonly employed tunneling materials include azo dyes, organometallic compounds, and charged iron complex species, which enable deeper electron access within enzymes or biological materials used in biosensors. Similarly, these effects enhance the electron-harvesting capability of supercapacitors, promoting energy storage efficiency.^55^

Typically, coating the active PGE surface results in an electron-blocking effect, reducing the electrode’s current value.^18^ However, the incorporation of UiO-66 and MOF-199 into PVDF nanofibers demonstrates the potential for electron tunneling and host effects, warranting further exploration. Several high-performance supercapacitors leveraging such nanofiber-based systems have been documented, suggesting significant potential for performance enhancements.^56,57^ Notably, UiO-66 exhibits higher chemical stability than MOF-199, providing long-term durability in electrochemical applications. The high surface area and porosity of UiO-66 offer more active sites, enhancing electrode performance. Additionally, its stable structure and well-arranged metal centers increase electrical conductivity, making UiO-66 more effective for energy storage and conversion applications. These characteristics establish UiO-66 as a superior material for electrochemical devices such as supercapacitors and batteries.^58^

When examining the Nyquist plots, linear spectra were observed, and the best fitting circuit was found as R1([R2Q1]C1)C2 for MOF-199 and R1([R2Q1]C1) for UiO-66, indicating Warburg’s impedance process. The circuit elements represent *R*_ct_ (charge transfer resistance), *C* (capacitance), and *Q* (Constant phase element), with the corresponding parameter values shown in Figure 7e. UiO-66 exhibited excellent fit with the proposed circuit model, yielding a chi-square value of 0.0012, compared to 0.0096 for MOF-199. The closer a chi-square value is to zero, the better the fit of the model.

The Nyquist plots in Figure 7d also reveal differences in charge transfer resistance and Warburg impedance for MOF-199 and UiO-66 electrodes. A higher slope in the Warburg region correlates with improved charge transfer capabilities. Conversely, a lower slope indicates increased inner resistance, hindering charge transfer. In the given Figure 7d according to the Warburg type of impedance, without a semicircle occurrence the slope of the line enlightens the charge resistance properties. If, the slope is higher it means the charge transfer is facilitated, besides as the slope of the Warburg spectra decreases this means the inner resistance has increased and the charge transfer is prevented. The UiO-66/PVDF/PGE electrode, exhibiting a steeper slope compared to the MOF-199/PVDF/PGE electrode, demonstrates higher charge transfer ability, consistent with the CV results.^19,59^ Previous studies support these findings, showing that UiO-66 has lower internal resistance^60^ and higher conductivity compared to MOF-199,^61^ further validating its suitability for advanced energy storage applications.

### 3.6 Supercapacitance Measurements

Following the characterization of the developed nanofibers using CV and EIS techniques, it was determined that UiO-66 and MOF-199 provided significant electrochemical contributions to the composite nanofiber structure. Then, the supercapacitive performance of nanofibers at various scanning speeds was evaluated with the corresponding specific capacitance values (*C*_s_) in 0.1 M KOH solution presented in Table 2. The “threshold good” values for supercapacitors vary depending on the type of material used. For carbon-based materials, this range typically falls between 100 and 300 F/g, with materials such as activated carbon and semiconducting carbon nanotubes achieving capacities up to 250 F/g. Supercapacitors employing ionic liquid or polymer electrodes often exceed 300 F/g. In summary, the “threshold good” values vary depending on the material used, but generally target a value between 100 and 300 F/g.^62^ In this context, the findings highlighted the remarkable integration and compatibility between UiO-66, MOF-199, and the PVDF matrix, as evidenced by the high *C*_s_ values achieved.

**Table 2:** *C*_s_ Values of the MOF-199/PVDF, UiO-66/PVDF Modified PGE Electrodes for Different Scan Rates

	specific capacitances of electrodes (F/g)	
scan rate (mV/s)	MOF-199/PVDF/PGE	UiO-66/PVDF/PGE
5	825.65	651.70
10	933.19	1619.26
50	766.86	1192.95
100	662.58	1016.32
250	536.36	821.34

**Figure 8:**
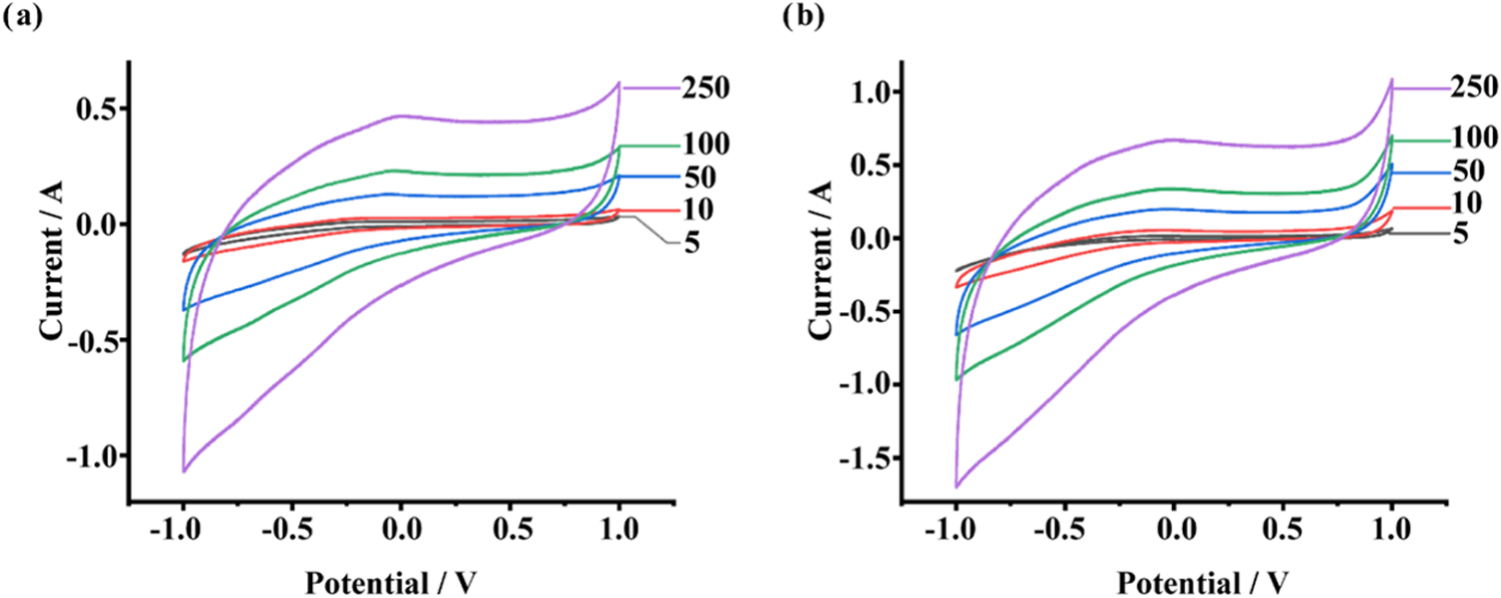
CV curves of (a) MOF-199/PVDF/PGE (b) UiO-66/PVDF/PGE electrodes at scan rate from 5 to 250 mV/s.

An important aspect of this arrangement is the role of the PVDF nanofiber as a supporting framework. In Figure 8, the current–potential results are overlapped and the best *C*_s_ values obtained from different scan rate measurements are shown for each electrode configuration. Examination of the peak resolutions and current values, alongside the corresponding *C*_s_ values in Table 2, revealed that the overlapping areas of the current–potential curves increased with higher scan rates, as expected. At lower scan rates, electrolyte ions had sufficient time to diffuse into the porous structure of the electrode material, enabling full access to the internal surface area. This facilitated maximum charge storage through both electrical double-layer capacitance (EDLC) and pseudocapacitance mechanisms. Conversely, at higher scan rates, ion diffusion was restricted due to time constraints, limiting the utilization of the electrode’s internal surface area. Consequently, only the outermost or most easily accessible regions contributed to charge storage.^63^

The results demonstrated that both electrodes exhibited favorable *C*_s_ values across all scan rates, with particularly notable performance at 10 mV/s (Table 2). At this scan rate, the UiO-66/PVDF/PGE electrode achieved a specific capacitance of 1619.26 F/g, while the MOF-199/PVDF/PGE electrode reached 933.19 F/g. These findings underscore the potential of the UiO-66 and MOF-199 composites within the PVDF matrix for advanced supercapacitor applications.

### 3.7 Long-Term Measurements

**Figure 9:**
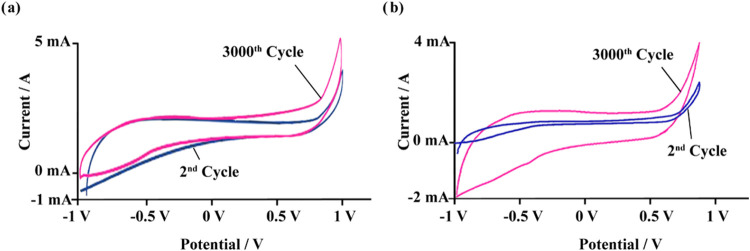
Cyclic stability of the (a) MOF-199/PVDF/PGE and (b) UiO-66/PVDF/PGE electrodes.

**Figure 10:**
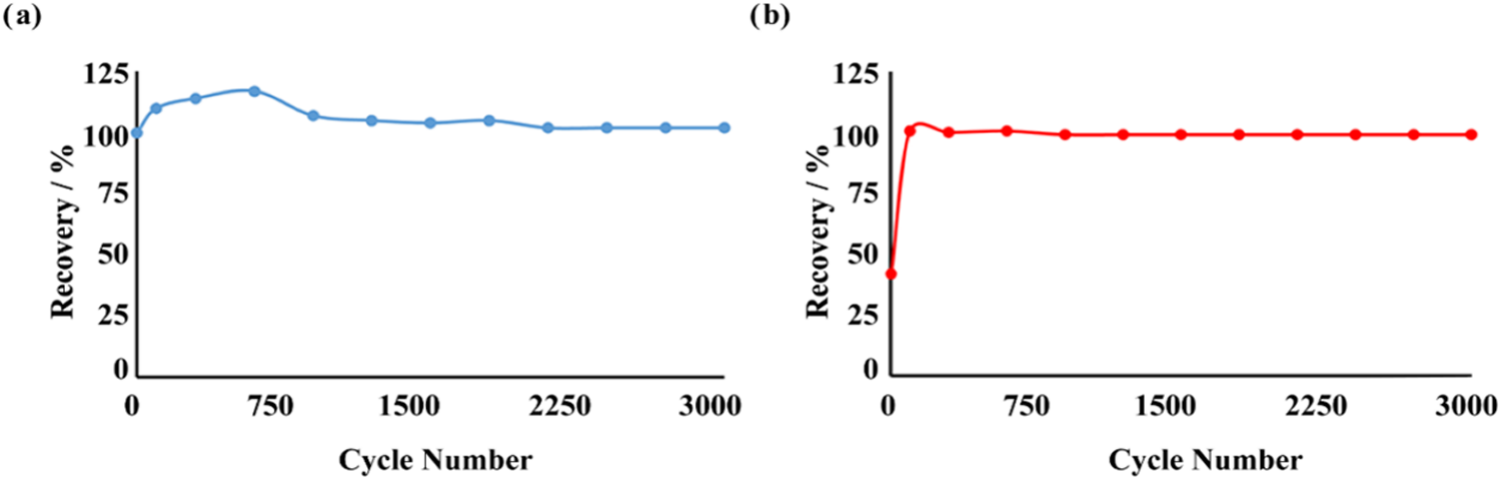
*C*_s_ recovery rates in long-term measurements of (a) MOF-199/PVDF/PGE and (b) UiO-66/PVDF/PGE.

Figure 9 illustrates the charge–discharge cycling behavior, showcasing the long-term specific capacitance (*C*_s_) stability of both electrodes, while Figure 10 highlights the *C*_s_ recovery rates observed during long-term measurements. According to the *C*_s_ retention data recorded every 250 cycles, the MOF-199/PVDF/PGE electrode stabilized after 750 cycles, achieving a long-term *C*_s_ retention rate of 102.04%. Similarly, the UiO-66/PVDF/PGE electrode stabilized after 600 cycles, with a long-term *C*_s_ retention rate of 99.16% (Figure 10). Despite the notable performance of MOF-199, its relatively lower stability compared to UiO-66 makes it susceptible to degradation under moisture or acidic/basic conditions, potentially impacting the composite’s long-term performance. Nevertheless, the flexibility of PVDF helps mitigate some of these limitations by enhancing structural integrity. UiO-66, in contrast, exhibits superior stability, enabling it to withstand harsher environments and maintain consistent performance in electrochemical devices. The robust Zr–O bonds within the UiO-66 framework prevent collapse or leaching, thereby improving the composite material’s long-term stability. Additionally, the closed system used for the measurements ensured no solution loss during the experiments, maintaining reliable results throughout the testing period.^18,19^ Collectively, these findings highlight the potential of MOF-199/PVDF/PGE and UiO-66/PVDF/PGE nanofibers as high-performance supercapacitor materials, demonstrating outstanding stability, conductivity, and energy storage capabilities.

The superior performance of these composites can be attributed to the inherent properties of UiO-66 and MOF-199, which exhibit high electrochemical activity and enhanced conductivity due to their large surface area and well-defined porosity. The integration of the PVDF polymer matrix plays a crucial role in achieving long-term electrode stability, aligning with findings from other studies on MOF-based composites. UiO-66′s high stability and compatibility with PVDF nanofibers ensure uniform dispersion within the PVDF matrix, maximizing the exposure of active surface areas for electrochemical or catalytic activity. This synergy results in enhanced electrochemical performance, including higher specific capacitance and better cycle stability. The robust Zr_6_ clusters of UiO-66 further contribute to its durability in harsh environments, improving the composite’s overall stability under extended electrochemical cycling. MOF-199, typically based on zinc or copper, can also be effectively integrated into PVDF nanofibers using electrospinning or solution-casting techniques. The nanofibers provide a scaffold with a large surface area for MOFs to anchor onto, enhancing stability and charge transport. However, the relatively softer structure of MOF-199 poses challenges in maintaining integrity under harsh conditions, especially during long-term electrochemical cycling.^64,65^ The results clearly demonstrate that significant improvement in long-term electrochemical stability for supercapacitor applications is possible by appropriately incorporating UiO-66 and MOF-199 into the structure of PVDF nanofiber.

The *C*_s_ values of materials examined in recent studies are summarized in Table 3, highlighting the influence of nanofiber selection. PVDF-MOF composites demonstrated two- to 3-fold higher F/g values compared to Polypyrrole (PPy), UiO-66 and cotton-based hybrid structures, and UiO-66 coated with polydopamine (PDA).^12^ Although Mn_2_O_3_@MnO_2_ composite nanofibers exhibit superior cyclic stability, their *C*_s_*p* values could be further enhanced by incorporating PVDF into their structure.^66^ This remarkable stability arises from the strong interaction between the MOF-199 and UiO-66 frameworks and the PVDF matrix. Hydrogen bonding between the F-rich PVDF structure and the O-rich MOF frameworks enhances mechanical integrity and mitigates structural degradation during prolonged cycling.^67^ Furthermore, the intrinsic properties of MOF-199 and UiO-66, such as their high surface area, redox-active sites, and efficient ion transport pathways, significantly contribute to their exceptional electrochemical performance. These findings demonstrate the critical role of MOF-PVDF composites in advancing the field of high-performance supercapacitors.

**Table 3:** Comparison of *C*_s_ Values Obtained from the Present Study and the Literature

supercapacitor composition	specific capacitance value (F/g)	cycle number	measurement medium	refs
UiO-66/PVDF	1619.26	3000	0.1 M KOH	(present study)
MOF-199/PVDF	933.19	3000	0.1 M KOH	(present study)
polypyrrole (PPy), UiO-66 and cotton based hybrid structure	565	500	1 M H_2_SO_4_ and 0.4 M hydroquinone	(7)
Zr-MOF (UiO-66 derivative)	811	2000	6 M KOH	(68)
UiO-66 coated with polydopamine (PDA)	822.04	4000	6 M KOH	(12)
conversion of MOF-199 to copper–copper oxide and carbon based nanostructures (Cu–Cu_2_O–CuO/C) by pyrolysis method	750	3000	6 M KOH	(42)
Mn_2_O_3_@MnO_2_ composite nanofibers	225	5000	0 1 M Na_2_SO_4_	(66)
UiO-66 and ZrO_2_ composites	913.8	3000	6 M KOH	(11)

## 4 Conclusions

The one-step electrospinning process was successfully utilized to fabricate UiO-66/PVDF/PGE and MOF-199/PVDF/PGE nanofiber electrodes. The synergistic interaction between PVDF polymers and MOFs resulted in composite nanofibers exhibiting excellent specific capacitance, low resistivity, and remarkable cycling stability. The UiO-66/PVDF/PGE electrode achieved an impressive specific capacitance of 1619.26 F/g at 1 A/g, retaining 99.16% of its initial capacitance after 3000 cycles. Similarly, the MOF-199/PVDF/PGE electrode demonstrated a specific capacitance of 933.19 F/g, retaining 102.04% of its initial capacitance over the same period, showcasing exceptional durability for long-term applications.

Thermal and mechanical analyses further corroborated the stability of these composites. The MOF-199/PVDF system offered enhanced flexibility and strain tolerance, while the UiO-66/PVDF composite exhibited superior mechanical strength. These findings highlight the potential of integrating MOFs into polymer nanofiber matrices as a pathway to developing advanced, high-performance electrode materials for next-generation supercapacitors.

## Supporting Information Available

Schematic representation of the nanofiber structure of MOF-199/PVDF (Figure S1); and PXRD Analysis section, and PXRD pattern of experimental (red and purple) and simulated from CIFs (blue and turquoise) for UiO-66/PVDF, MOF-199/PVDF (Figure S2) (PDF)
