# Supplementary material for: One-Step Fabrication of UiO-66/PVDF/PGE and MOF-199/PVDF/PGE Electrode for High-Performance Supercapacitors
Source: ACS Omega. 2025 Mar 28;10(18):18583–95. doi: 10.1021/acsomega.4c11211 (PMC12079280; doi:10.1021/acsomega.4c11211)
Supplement: Supplementary file 1 — ao4c11211_si_001.pdf [file ao4c11211_si_001.pdf]

Supplementary Information:

One-Step Fabrication of UiO-66/PVDF/PGE and MOF-199/PVDF/PGE Electrode for High-Performance Supercapacitors

Ozay Eroglu[1], H. Sevval Dere[1], Afike Ayca Ozen[2], Sema Aslan[2], Siti Nadiah Abdul Halim[1,3], Ugur Erkarslan[4], Hulya Kara Subasat[1,]*

[1]Department of Energy, Molecular Nano-Materials Laboratory, Mugla Sıtkı Kocman University, Mugla, Turkey

[2]Department of Chemistry, Mugla Sıtkı Kocman University, Muğla, Turkey

[3]Department of Chemistry, Faculty of Science, Universiti Malaya, Kuala Lumpur, Malaysia

[4]Department of Physics, Molecular Nano-Materials Laboratory, Mugla Sıtkı Kocman University, Mugla, Turkey

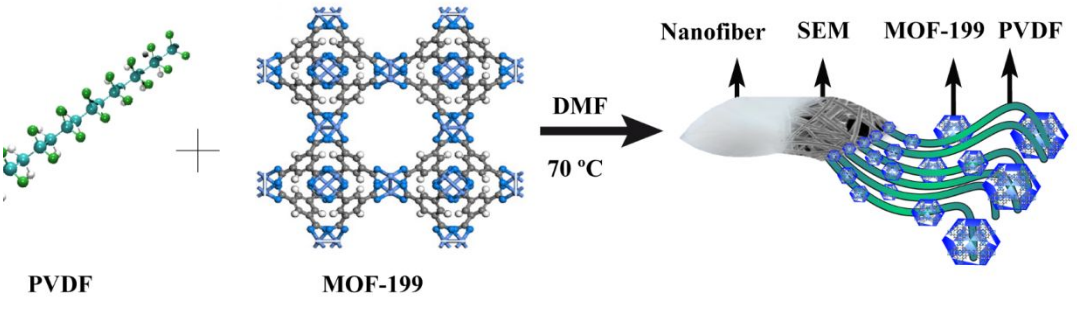

Figure S1. Schematic representation of the nanofiber structure of MOF-199/PVDF.

PXRD Analysis

Figure S2 displays a comparison between the PXRD patterns of the synthesized MOFs (shown in red and purple) and those simulated from CIFs (Crystallographic Information File) (shown in blue and turquoise). The patterns demonstrate a close match, suggesting that the bulk materials produced have high crystallinity and are representative of single crystals. It is important to note, however, that the characteristic peak of MOF-199 (occurring at 2θ = 6.9) was attributed to the difference in the copper (II) precursor used[1]. Peak values are compatible with the literature[2,3]

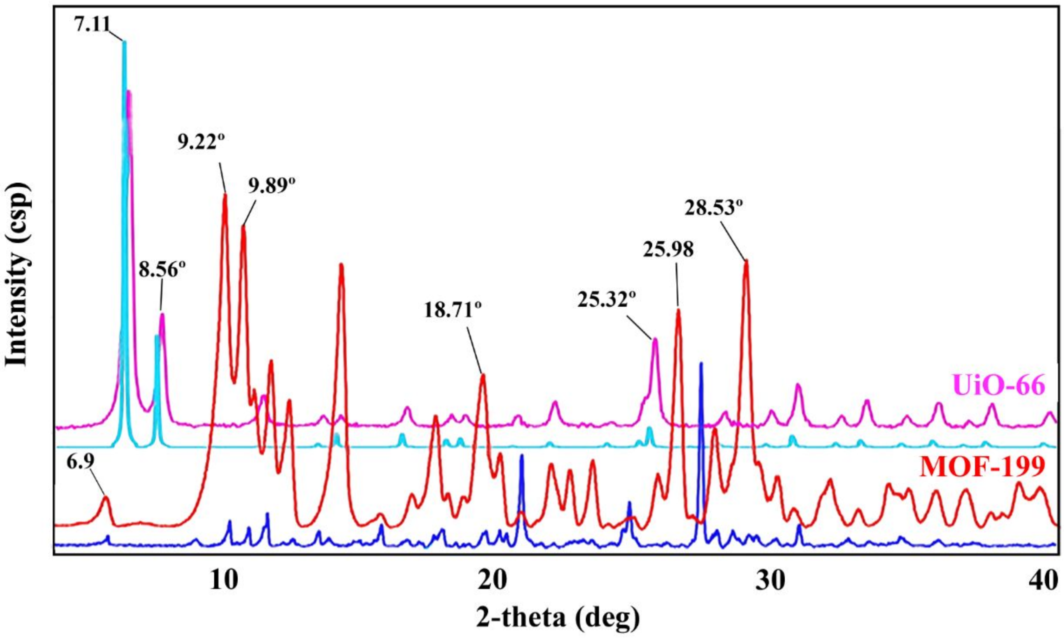

Figure S2. PXRD pattern of experimental (red and purple) and simulated from CIFs (blue and turquoise) for UiO-66/PVDF, MOF-199/PVDF.

References

1. Liu W, Huang C, Jin X. Tailoring the grooved texture of electrospun polystyrene nanofibers by controlling the solvent system and relative humidity. *Nanoscale Res Lett* 2014; 9: 1–10.
2. Aghajanzadeh M, Zamani M, Molavi H, et al. Preparation of Metal–Organic Frameworks UiO-66 for Adsorptive Removal of Methotrexate from Aqueous Solution. *J Inorg Organomet Polym Mater* 2018; 28: 177–186.
3. Liu N, Yao Y, Cha JJ, et al. Functionalization of silicon nanowire surfaces with metal-organic frameworks. *Nano Res* 2012; 5: 109–116.
